# Job burnout and perceived humanistic management: a cross-sectional study of hospital logistics support staff

**DOI:** 10.3389/fpsyg.2026.1921755

**Published:** 2026-09-21

**Authors:** Yuming Gao, Jiayi Wang, Huina Wang, Qingzhu Ding, Hansong Fan, Jin Gao, Xiaolan You, Peng Qian

**Affiliations:** 1 The Affiliated Taizhou People's Hospital of Nanjing Medical University, Taizhou, Jiangsu, China; 2 Affiliated Hospital of Yangzhou University, Yangzhou, China

**Keywords:** hospital logistics staff, humanistic management, job burnout, logistics management, occupational mental health

## Abstract

**Introduction:**

Hospital logistics support staff bear essential auxiliary tasks in hospitals, and inadequate perceived humanistic management tends to aggravate their job burnout, which has not been fully explored in existing research.

**Methods:**

This cross-sectional study recruited 205 hospital logistics support staff to investigate the correlation between perceived humanistic management and job burnout as well as their influencing factors. Questionnaires including the MBI-GS and a self-developed humanistic management perception scale were used for data collection, with univariate analysis, Pearson correlation analysis and multiple linear regression adopted for statistical processing.

**Results:**

Educational attainment, annual income and daily working hours independently predicted all burnout dimensions; higher education and income alleviated burnout, while prolonged working hours exacerbated it. With burnout treated as a covariate, education, tenure, daily working hours and burnout were consistent predictors across all four humanistic management subdimensions, whereas demographic and occupational factors exerted heterogeneous effects on partial dimensions. All subdimensions of humanistic perception showed significant moderate negative correlations with burnout.

**Discussion:**

This study supplements empirical evidence on occupational mental health of understudied logistics support staff. Hospitals can mitigate burnout among logistics support staff by controlling daily workload, establishing equitable salary systems and implementing tailored humanistic management strategies.

## 1 Introduction

Occupational burnout is a chronic psychological stress syndrome induced by prolonged work-related strain, which is prevalent in high-intensity occupational scenarios characterized by persistent work pressure, excessive workload and intensive emotional consumption (Obeng Nkrumah et al., 2025). In 2019, the World Health Organization (WHO) officially incorporated occupational burnout into the 11th revision of the International Classification of Diseases (ICD-11), defining it as a work-related occupational phenomenon rather than an independent medical condition. This syndrome is predominantly manifested through three core dimensions: emotional exhaustion, cynical detachment, and diminished professional efficacy (Franca et al., 2023). As a prevalent occupational psychological hazard in the healthcare industry, burnout not only impairs employees’ physical and mental well-being but also triggers a series of adverse organizational consequences, thereby undermining the stability and operational quality of healthcare institutions.

Hospital logistics support staff, including logistics management personnel, environmental cleaning personnel, security staff, laundry attendants, equipment maintenance technicians, supply administrators, and cafeteria workers, constitute an indispensable foundational workforce that guarantees the continuous and standardized operation of hospital healthcare services. Nevertheless, this group commonly endures considerable occupational stress and exhibits a high susceptibility to job burnout, with logistics hospital staff reporting comparable or even higher burnout risks than clinical personnel due to long-term repetitive work and insufficient organizational attention (Goel et al., 2023). Severe burnout among logistics personnel may lead to deteriorated psychological and physical health, inhibited career development, declined work efficiency and service quality, increased institutional labor costs, and elevated operational and safety risks within medical settings (Clinchamps et al., 2021). For example, burnout-induced negative work status and decreased vigilance may compromise equipment maintenance quality, environmental sanitation management and material supply accuracy, further predisposing hospitals to nosocomial infection hazards and operational disorders (Matern, 2011). Regrettably, due to the hidden and auxiliary nature of logistics work, the occupational psychological health and burnout problems of support staff have long been underestimated and neglected in hospital management and academic research, resulting in insufficient targeted attention and intervention. In this context, it is of great practical significance to explore the key influencing factors of job burnout among hospital logistics employees.

Humanistic management within hospitals is a management philosophy and practical approach centered on the principle of “people first.” It systematically integrates the spirit of medical humanism—encompassing respect, empathy, understanding, and compassionate care—into all aspects of hospital operations. This model emphasizes the protection of the values, dignity, emotional well-being, and professional or personal needs of all stakeholders within the health care environment, such as patients (Chen et al., 2024; Leong et al., 2025; Lv et al., 2025), clinical staff (Shanafelt et al., 2017; Dyrbye and Shanafelt, 2016; West et al., 2016), and administrative support personnel (Blake, 1987). By fostering a culture of mutual respect and holistic care, the aim of this model is to promote humane, high-quality, and sustainable development in health care services (Blake, 1987).

The perception of humanistic management refers to employees’ subjective cognitive evaluation and emotional experience regarding the implementation of humanistic systems, supportive practices and organizational atmosphere within hospital management and service processes (Zhao et al., 2025). Affected by individual heterogeneity, environmental conditions and organizational management differences, logistics support staff may demonstrate varying degrees of decline in humanistic management perception.

Optimized humanistic institutional arrangements and supportive organizational environments have been verified to effectively buffer occupational stress and reduce negative psychological outcomes among healthcare employees (Rider et al., 2018; Li et al., 2025). Nevertheless, the interactive relationship between humanistic management perception and job burnout remains poorly understood among hospital logistics populations. It remains empirically unclear whether low humanistic management perception serves as a risk factor for aggravated burnout, or conversely, whether persistent burnout symptoms erode employees’ positive perception of organizational humanistic care, thereby forming a potential bidirectional vicious cycle (Golu et al., 2022). The absence of empirical evidence concerning their interactive mechanism has created a notable research gap, limiting the development of targeted psychological intervention and refined human resource management strategies for hospital logistics teams.

Existing studies regarding hospital humanistic management predominantly focus on its effects on medical quality and patient satisfaction, with research subjects concentrated on clinical nursing staff (Guo et al., 2022). Prior studies have verified that optimized humanistic management can effectively alleviate occupational stress and reduce burnout levels among nurses (Lv et al., 2025). However, due to substantial differences in job attributes, salary structure, social identity and occupational environment between clinical nurses and logistics support staff, existing research conclusions cannot be generalized to logistics populations (Delpy et al., 2025; Galanis et al., 2023). To date, empirical studies focusing on the correlation between humanistic management perception and job burnout among hospital logistics employees remain scarce, and no published literature has explored their potential bidirectional interactive relationship. Given the unique occupational characteristics and high burnout risk of logistics support staff, targeted investigation is urgently required.

The exploration of the association between humanistic management perception and job burnout among hospital logistics support staff possesses profound theoretical and practical value. Theoretically, it complements the empirical evidence of organizational psychology and healthcare human resource management, enriches the research system of occupational mental health in non-clinical medical populations, and provides new perspectives for social equity research in hospital workforce management (Claponea and Iorga, 2023). Practically, this study contributes to improving the overall operational efficiency of healthcare institutions and offers empirical support for constructing a sustainable, humanized and refined hospital logistics management system (Claponea and Iorga, 2023). For hospital administrative practice, this research helps managers systematically understand the current status of humanistic management perception and job burnout among grassroots logistics support staff, accurately identify potential psychological management risks, and clarify the interactive mechanism between organizational management atmosphere and employee psychological status. The findings can provide targeted, evidence-based optimization schemes for hospital logistics human resource allocation, humanistic system improvement, and staff psychological health intervention, which is conducive to stabilizing the logistics workforce, reducing institutional operational risks, and promoting the high-quality development of modern hospital logistics management (Ju et al., 2026).

Based on relevant theoretical foundations and existing research findings, this study proposes the following hypotheses: humanistic management perception is significantly and negatively correlated with job burnout among hospital logistics support staff; moreover, a bidirectional interactive and mutually aggravating relationship exists between reduced humanistic management perception and elevated occupational burnout. Accordingly, this study aims to investigate the current status of humanistic management perception and job burnout among hospital logistics support staff, analyze their correlation, and further verify the bidirectional interactive mechanism between the two variables.

## 2 Source of data and methods

### 2.1 Research subjects

In this study, the logistics support staff of Taizhou People’s Hospital, affiliated with Nanjing Medical University, were taken as the research subjects. Participants were recruited by stratified random sampling. Stratification was performed strictly according to job category, and each stratum was mutually exclusive: security services, catering services, environmental cleaning, logistics management personnel, and other logistics support posts. All eligible staff on the payroll within each stratum were included in the sampling frame, with a complete roster provided by the hospital logistics department to ensure every qualified individual had equal probability of being selected, avoiding omission of any population unit and sampling selection bias. The inclusion criteria were as follows: (1) Full-time employment in a logistics-related hospital department for ≥1 year, (2) Direct involvement in logistics support work, (3) Weekly working hours ≥30 h, (4) Voluntary participation in the questionnaire survey. The exclusion criteria were as follows: (1) non-logistics staff, (2) personnel stationed from external units but not directly managed by the hospital, (3) individuals on long-term sick leave/leave, (4) those concurrently participating in other occupational mental health intervention programs, and (5) individuals with a history of severe mental illness or who were currently taking related medications. G*Power 3.1 was used to pre-calculate sample size for multiple linear regression. With f^2^ = 0.15, *α* = 0.05, power = 0.80 and 12 predictors, the minimum valid sample was 139. Factoring in 20% invalid questionnaires, we aimed to recruit 174 participants, and a total of 220 participants were enrolled via stratified sampling. The study was registered at Chictr.Org.cn: ChiCTR 2500110473, and it was approved by the Ethics Committee of Taizhou People’s Hospital affiliated with Nanjing Medical University (Approval No. LSKY2025-108-01). Eligible hospital logistics personnel were enrolled in the study after informed consent was obtained and all methods were performed in accordance with the relevant guidelines.

### 2.2 Research tools

#### 2.2.1 General information questionnaire

General Information Questionnaire: This self-designed questionnaire collected participants’ baseline data covering demographic features (gender, age, marital status, education level), annual personal income, work schedule (daily working hours, Scheduling type), and occupational information including employment type, specific job position, years of service, and performance appraisal. The performance appraisal item was a binary qualitative indicator simply classifying subjects into groups with or without institutional appraisal. These data included age, sex, marital status, average annual income, daily working hours, scheduling type, performance appraisal, employment type, job position, years of service, and highest education level, among other details.

#### 2.2.2 Hospital humanistic management perception questionnaire

Questionnaires for perceived humanistic management within hospitals were designed via Wenjuanxing platform and then printed into paper versions for on-site distribution and completion by participants. Preliminary items were extracted from validated published scales (Blake, 1987; Perez-Fuentes et al., 2019), and supplemented through on-site staff interviews for localized revision. Two hospital management experts and one statistics expert were invited to evaluate the clarity and content validity of the items, leading to the deletion of ambiguous or redundant questions. All items adopt 5-point Likert scoring (1 = strongly disagree, 2 = disagree, 3 = neutral, 4 = agree, and 5 = strongly agree) without reverse questions. The final scale consists of four dimensions derived from humanistic management theory and organizational support theory (Blake, 1987), referencing existing healthcare humanization scale frameworks: Work Collaboration and Respect Perception (WCR), Work Support and Career Development Perception (SCD), Management System and Care Perception (MSC), and Cultural and Value Identity Perception (CVI). A pilot test was conducted with 30 logistics support staff members, and the reliability of the scale was analysed using SPSS software. In this study, the overall Cronbach’s *α* coefficient for the Humanistic Management Perception Questionnaire was 0.955, while the coefficients for the four dimensions ranged from 0.753 to 0.889.

#### 2.2.3 Maslach burnout inventory

The job burnout questionnaire used in this study was the Maslach Burnout Inventory-General Survey (MBI-GS), which was developed by Maslach et al. (2001) and subsequently translated and revised by Li et al. (2001). The MBI-GS (16-item general survey version) was selected rather than the 22-item MBI-HSS in the present study, since our subjects were non-clinical hospital logistics support staff, which matches the applicable population of MBI-GS. The scale consists of three dimensions: Emotional Exhaustion (EE, 5 items), Depersonalization (DP, 4 items), and Personal Accomplishment (PA, 6 items). All the items are rated on a 7-point Likert scale. EE and DP are positively scored, whereas PA is reverse scored, with anchors as follows: 0 = “Never,” 1 = “A few times a year or less,” 2 = “Once a month or less,” 3 = “A few times a month,” 4 = “Once a week,” 5 = “A few times a week,” and 6 = “Every day.” A pilot test was similarly conducted with 30 participants. Reliability analysis indicated a Cronbach’s *α* coefficient of 0.931 for the entire scale, with the coefficients for the three dimensions ranging from 0.751 to 0.881. Formal license to administer MBI-GS for 205 participants was purchased from Mind Garden, Inc. after the final valid sample size was confirmed in this research; the license is valid for 3 years starting October 21, 2025. Full scale items are not presented in the manuscript to comply with the publisher’s copyright regulations.

### 2.3 Data collection

Data were collected by distributing paper-based questionnaires. The researcher printed the questionnaires, which consisted of three parts—general information, the Humanistic Management Perception Questionnaire, and the Maslach Burnout Inventory (MBI-GS)—and distributed them to the study subjects. All participants completed the survey after providing informed consent and meeting the inclusion and exclusion criteria. Prior to completing the questionnaire, the purpose, methods, and precautions of the survey were explained to the participants. Respondents finished questionnaires independently on site; researchers timely checked for obvious missing items during completion and prompted participants to supplement missing content immediately, effectively raising the valid questionnaire rate. The whole filling process took approximately 45 min. Upon completion, all the questionnaires were collected and individually checked for completeness, and any invalid questionnaires were discarded. The valid data were then entered into an Excel spreadsheet.

### 2.4 Statistical analysis

Data analysis was performed using SPSS 25.0 statistical software. Internal consistency reliability (Cronbach’s *α*) was used to assess the reliability of the scales, with α coefficients calculated for both the total scales and their respective subdimensions; an α coefficient >0.7 indicated good reliability. Categorical data were expressed as proportions (%). Normally distributed measurement data were presented as the mean ± standard deviation. The Shapiro–Wilk test was adopted to verify the normal distribution of continuous variables. Pearson correlation analysis was applied to examine the correlations between study variables. One-way ANOVA was used for univariate intergroup comparisons. Variables with *p* < 0.05 in univariate analyses were further included in multiple linear regression models to identify independent influencing factors. Variance inflation factor (VIF) was calculated to test multicollinearity among independent variables, and the Durbin–Watson test was used to evaluate residual independence. A two-tailed *p* value < 0.05 was considered to indicate statistical significance.

## 3 Results

### 3.1 General information of the research subjects

A total of 220 questionnaires were distributed in this study, and 205 valid questionnaires were collected, yielding a response rate of 93.18%. The general information of the research subjects is presented in Table 1. Job burnout was stratified into three groups based on weighted total scores (0–6) following the Ahola standard: weighted score = (0.4 times) mean EE (+0.3 times) mean DP (+0.3 times) reversed mean PA. Cut-off values: no burnout (0–1.49), mild burnout (1.50–3.49), severe burnout (≥3.50).

**Table 1:** Basic information of the research subjects.

Parameter	Number of cases (%)
Gender	
Male	111 (54.15)
Female	94 (45.85)
Age (years)	
<30	44 (21.46)
30–39	82 (40.00)
40–49	51 (24.88)
50–59	28 (13.66)
Marital status	
Unmarried	84 (40.98)
Married	108 (52.68)
Divorced or Widowed	13 (6.34)
Educational attainment	
Junior high school and below	38 (18.54)
High school/Technical secondary school	81 (39.51)
Junior college	54 (26.34)
Bachelor’s degree and above	32 (15.61)
Annual income (ten thousand RMB)	
<5	74 (36.10)
5–10	119 (58.05)
>10	12 (5.85)
Daily working hours (h)	
≤8	154 (75.12)
>8	51 (24.88)
Scheduling type	
Fixed weekend	86 (41.95)
Flexible leave	119 (58.05)
Employment type	
Outsourced staff	140 (68.29)
Formal employees	65 (31.71)
Job position	
Security Services	35 (17.07)
Catering services	38 (18.54)
Environmental Cleaning	48 (23.41)
Logistics management	37 (18.05)
Other Logistics Support Posts	47 (22.93)
Years of services (y)	
1–3	30 (14.64)
4–5	121 (59.02)
≥6	54 (26.34)
Performance appraisal	
No	87 (42.44)
Yes	118 (57.56)
Job burnout	
No burnout	84 (40.98)
Mild burnout	54 (26.34)
Severe burnout	67 (32.68)

### 3.2 Univariate analysis of occupational burnout and its dimensions in hospital logistics support staff

Univariate analyses stratified by demographic characteristics were conducted for burnout subscales (Table 2). Marital status and educational attainment were significantly correlated with EE, DP, and PA (all *p* < 0.05).

**Table 2:** Univariate analysis of burnout by demographic characteristics.

Parameter	EE (x̄±SD)	DP (x̄±SD)	PA (x̄±SD)
Gender			
Male (*n* = 111)	13.41 ± 10.94	9.77 ± 7.83	23.12 ± 10.03
Female (*n* = 94)	13.40 ± 10.73	8.98 ± 7.22	21.98 ± 10.33
*t* value	0.01	0.75	0.80
*p* value	0.99	0.45	0.43
Ages			
<30 Years (*n* = 44)	13.77 ± 11.61	10.20 ± 8.68	22.23 ± 10.77
30–39 Years (*n* = 82)	12.73 ± 10.83	8.82 ± 7.35	24.17 ± 9.92
40–49 Years (*n* = 51)	13.73 ± 9.11	9.75 ± 6.30	20.43 ± 9.67
50–59 Years (*n* = 28)	14.25 ± 12.69	9.29 ± 8.52	22.50 ± 10.49
*F* value	0.19	0.36	1.46
P value	0.90	0.78	0.23
Marital status			
Unmarried (*n* = 84)	15.68 ± 11.48	11.20 ± 8.24	20.56 ± 10.57
Married (*n* = 108)	10.90 ± 9.83	7.62 ± 6.74	24.92 ± 9.29
Divorced or Widowed (*n* = 13)	19.62 ± 9.00	12.69 ± 5.56	16.46 ± 9.67
*F* value	7.33	7.03	7.30
*p* value	0.00	0.00	0.00
Educational attainment			
Junior high school and below (*n* = 38)	20.24 ± 8.41	13.71 ± 7.21	17.37 ± 9.45
High school/Technical secondary school (*n* = 81)	15.64 ± 10.37	10.79 ± 6.90	21.32 ± 9.69
Junior college (*n* = 54)	9.67 ± 10.88	7.28 ± 7.45	24.56 ± 10.27
Bachelor’s degree and above (*n* = 32)	5.97 ± 7.33	4.41 ± 5.77	28.72 ± 8.18
*F* value	16.45	13.13	9.35
*p* value	0.00	0.00	0.00

Subsequent univariate analyses of burnout across working schedule indicators are presented in Table 3 (*n* = 205). Average annual income, daily working hours, and scheduling type were significantly associated with EE, DP and PA (all *p* < 0.05).

**Table 3:** Univariate analysis of burnout by working schedule indicators.

Parameter	EE (x̄±SD)	DP (x̄±SD)	PA (x̄±SD)
Annual income (ten thousand RMB)			
<5 (*n* = 74)	22.59 ± 7.61	16.26 ± 4.88	14.66 ± 8.48
5–10 (*n* = 119)	7.44 ± 8.54	5.26 ± 5.71	27.82 ± 7.63
>10 (*n* = 12)	16.00 ± 6.52	8.33 ± 6.72	19.67 ± 9.16
*F* value	80.30	91.81	62.06
*p* value	0.00	0.00	0.00
Daily working hours			
≤8 h (*n* = 154)	11.23 ± 10.54	8.02 ± 7.20	24.30 ± 9.65
>8 h (*n* = 51)	19.98 ± 8.87	13.61 ± 7.06	17.45 ± 10.00
*t* value	5.33	4.83	4.35
*p* value	0.00	0.00	0.00
Scheduling type			
Fixed weekend (*n* = 86)	16.98 ± 10.86	11.56 ± 7.44	20.40 ± 9.83
Flexible leave (*n* = 119)	10.83 ± 10.06	7.86 ± 7.27	24.18 ± 10.13
*t* value	4.17	3.56	2.68
*p* value	0.00	0.00	0.01

Results from univariate analyses of occupational attributes are displayed in Table 4 (*n* = 205). Employment type, job position, and performance appraisal were significantly associated with EE, DP and PA (all *p* < 0.01).

**Table 4:** Univariate analysis of burnout by occupational attributes.

Parameter	EE (x̄±SD)	DP (x̄±SD)	PA (x̄±SD)
Employment type			
Outsourced staff (*n* = 140)	15.58 ± 11.01	11.05 ± 7.44	20.80 ± 10.25
Formal employees (*n* = 65)	8.74 ± 8.77	5.88 ± 6.53	26.46 ± 8.85
*t* value	4.78	5.04	4.05
*p* value	0.00	0.00	0.00
Job position			
Security (*n* = 35)	16.09 ± 11.56	12.00 ± 7.96	19.89 ± 10.56
Dining services (*n* = 38)	17.82 ± 9.89	11.74 ± 6.68	19.53 ± 10.15
Cleaning (*n* = 48)	13.06 ± 10.90	8.94 ± 6.94	22.88 ± 10.20
Administrative management (*n* = 37)	6.89 ± 7.78	5.35 ± 6.31	27.38 ± 9.14
Other (*n* = 47)	13.34 ± 10.80	9.28 ± 8.14	23.04 ± 9.41
*F* value	6.03	5.03	3.77
*p* value	0.00	0.00	0.01
Years of services (y)			
1–3 years (*n* = 30)	15.03 ± 12.68	11.23 ± 9.69	21.47 ± 11.94
4–5 years (*n* = 121)	13.58 ± 10.73	8.78 ± 7.19	23.13 ± 9.27
≥6 years (*n* = 54)	12.13 ± 9.92	9.81 ± 6.91	22.02 ± 11.08
*F* value	0.73	1.38	0.44
*p* value	0.48	0.25	0.65
Performance appraisal			
No (*n* = 87)	18.54 ± 10.02	13.43 ± 6.87	19.20 ± 10.27
Yes (*n* = 118)	9.63 ± 9.81	6.45 ± 6.61	25.10 ± 9.35
*t* value	6.37	7.34	4.29
*p* value	0.00	0.00	0.00

### 3.3 Univariate analyses of perceived humanistic management dimensions

Scores were assigned to the perception of humanistic management and its various dimensions among logistics support staff to analyse the influencing factors. Univariate analyses for four subscales of humanistic management perception are presented in Table 5. Age, educational attainment, and Job burnout were significantly correlated with WCR, SCD, MSC, and CVI (all *p* < 0.05).

**Table 5:** Univariate analysis of humanistic management perception by demographics.

Parameter	WCR (x̄±SD)	SCD (x̄±SD)	MSC (x̄±SD)	CVI (x̄±SD)
Gender				
Male (*n* = 111)	39.62 ± 10.36	41.58 ± 11.11	41.68 ± 11.84	42.57 ± 11.43
Female (*n* = 94)	37.48 ± 10.03	40.13 ± 11.25	39.33 ± 14.00	41.69 ± 11.07
*t* value	1.50	0.92	1.28	0.55
*p* value	0.14	0.36	0.20	0.58
Ages				
<30 Years (*n* = 44)	32.34 ± 13.86	35.30 ± 14.47	32.27 ± 16.04	35.27 ± 15.46
30–39 Years (*n* = 82)	43.20 ± 4.73	45.90 ± 3.55	46.29 ± 4.08	46.40 ± 3.69
40–49 Years (*n* = 51)	37.65 ± 9.46	38.96 ± 13.40	37.71 ± 15.28	41.65 ± 11.83
50–59 Years (*n* = 28)	37.00 ± 11.02	38.68 ± 10.00	42.46 ± 11.53	41.54 ± 11.94
*F* value	13.43	11.62	15.17	10.82
*p* value	0.00	0.00	0.00	0.00
Marital status				
Unmarried (*n* = 84)	36.92 ± 11.61	40.17 ± 11.89	38.98 ± 13.86	40.44 ± 12.74
Married (*n* = 108)	39.83 ± 9.54	41.40 ± 11.20	41.16 ± 12.63	43.03 ± 10.46
Divorced or Widowed (*n* = 13)	39.85 ± 2.08	41.69 ± 4.31	46.46 ± 4.22	46.15 ± 2.82
*F* value	2.03	0.32	2.13	2.15
*p* value	0.13	0.73	0.12	0.12
Educational attainment				
Junior high school and below (*n* = 38)	24.79 ± 9.91	26.11 ± 11.13	22.21 ± 10.33	27.74 ± 11.44
High school/Technical Secondary school (*n* = 81)	38.43 ± 8.86	40.01 ± 9.53	39.99 ± 11.39	41.54 ± 10.35
Junior college (*n* = 54)	43.89 ± 2.34	47.50 ± 2.64	48.93 ± 1.87	48.63 ± 2.18
Bachelor’s degree and above (*n* = 32)	46.75 ± 3.18	49.66 ± 1.29	49.94 ± 0.35	49.97 ± 0.18
*F* value	69.37	72.00	89.63	59.58
*p* value	0.00	0.00	0.00	0.00
Job burnout				
No burnout (*n* = 84)	43.83 ± 4.44	46.75 ± 5.05	47.63 ± 4.93	48.01 ± 3.38
Mild burnout (*n* = 54)	35.59 ± 11.16	37.72 ± 12.56	36.44 ± 14.57	40.26 ± 11.99
Severe burnout (*n* = 67)	34.58 ± 11.66	36.16 ± 12.35	35.13 ± 14.34	36.37 ± 13.44
*F* value	22.30	24.21	26.67	26.20
*p* value	0.00	0.00	0.00	0.00

Subsequent univariate analyses of humanistic management perception are presented in Table 6 (*n* = 205). Annual income and daily working hours were significantly associated with WCR, SCD, MSC, and CVI (all *p* < 0.05). Scheduling type showed no significant correlation with WCR (*p* > 0.05), but was significantly correlated with SCD, MSC, and CVI (all *p* < 0.05).

**Table 6:** Univariate analysis of humanistic management perception by work schedule.

Parameter	WCR (x̄±SD)	SCD (x̄±SD)	MSC (x̄±SD)	CVI (x̄±SD)
Annual income (ten thousand RMB)				
<5 (*n* = 74)	37.27 ± 11.17	40.69 ± 11.11	40.38 ± 13.21	40.93 ± 11.94
5–10 (*n* = 119)	40.76 ± 9.07	42.94 ± 9.77	42.96 ± 11.05	44.09 ± 10.51
>10 (*n* = 12)	26.00 ± 0.00	22.17 ± 6.63	18.58 ± 5.16	30.67 ± 4.62
*F* value	13.93	22.93	23.85	9.14
*p* value	0.00	0.00	0.00	0.00
Daily working hours				
≤8 h (*n* = 154)	42.33 ± 6.72	44.68 ± 7.79	44.95 ± 9.03	45.82 ± 8.11
>8 h (*n* = 51)	27.49 ± 11.01	29.53 ± 12.14	27.45 ± 13.95	31.12 ± 12.22
*t* value	9.08	8.36	8.40	8.03
*p* value	0.00	0.00	0.00	0.00
Scheduling type				
Fixed weekend (*n* = 86)	37.80 ± 10.59	38.86 ± 11.18	38.50 ± 11.92	39.70 ± 11.66
Flexible leave (*n* = 119)	39.24 ± 9.99	42.39 ± 10.97	42.12 ± 13.41	43.95 ± 10.64
*t* value	0.99	2.26	2.00	2.71
*p* value	0.32	0.03	0.05	0.01

Results from univariate analyses of occupational attributes are displayed in Table 7 (*n* = 205). Job position, years of service, and performance appraisal were significantly associated with WCR, SCD, MSC, and CVI (all *p* < 0.05). Employment type showed no significant correlation with MSC (*p* > 0.05), but was significantly correlated with WCR, SCD and CVI (all *p* < 0.05).

**Table 7:** Univariate analysis of humanistic management perception by occupation.

Parameter	WCR (x̄±SD)	SCD (x̄±SD)	MSC (x̄±SD)	CVI (x̄±SD)
Employment type				
Outsourced staff (*n* = 140)	37.21 ± 10.83	39.81 ± 11.22	39.52 ± 13.14	40.74 ± 12.35
Formal employees (*n* = 65)	41.71 ± 8.09	43.28 ± 10.76	42.92 ± 12.12	45.23 ± 7.61
*t* value	3.31	2.08	1.77	3.19
*p* value	0.00	0.04	0.08	0.00
Job position				
Security (*n* = 35)	35.23 ± 2.21	36.54 ± 13.73	36.03 ± 14.12	37.60 ± 13.30
Dining services (*n* = 38)	38.74 ± 8.15	40.34 ± 9.14	40.03 ± 11.03	41.29 ± 10.22
Cleaning (*n* = 48)	41.27 ± 7.94	44.31 ± 8.70	44.40 ± 10.59	44.63 ± 8.91
Administrative management (*n* = 37)	46.46 ± 3.30	48.89 ± 2.25	49.54 ± 1.37	49.62 ± 1.14
Other (*n* = 47)	32.26 ± 11.04	34.87 ± 12.26	33.55 ± 15.17	37.89 ± 13.34
*F* value	14.92	13.16	12.37	9.07
*p* value	0.00	0.00	0.00	0.00
Years of services (y)				
1–3 years (*n* = 30)	31.33 ± 15.36	35.00 ± 15.02	32.07 ± 17.02	33.43 ± 15.40
4–5 years (*n* = 121)	38.40 ± 9.66	40.01 ± 11.37	40.31 ± 13.12	42.54 ± 11.21
≥6 years (*n* = 54)	43.22 ± 3.40	46.22 ± 3.70	46.00 ± 4.52	46.19 ± 3.57
*F* value	14.84	11.83	12.62	14.18
*p* value	0.00	0.00	0.00	0.00
Performance appraisal				
No (*n* = 87)	35.99 ± 11.77	38.71 ± 10.89	37.53 ± 12.19	38.64 ± 11.64
Yes (*n* = 118)	40.59 ± 8.48	42.53 ± 11.15	42.86 ± 12.99	44.76 ± 10.25
*t* value	3.10	2.45	2.98	3.91
*p* value	0.00	0.02	0.00	0.00

### 3.4 Multiple linear regression analysis of occupational burnout

Three separate multiple linear regression analyses were performed, with the three job burnout dimensions (EE, DP, PA) as dependent variables and demographic and work-related indicators as independent variables. Model diagnostics confirmed no residual autocorrelation (Durbin-Watson = 1.899–2.031), and all regression models were statistically significant (*p* < 0.001). The adjusted *R*^2^ values for EE, DP, and PA were 0.491, 0.550, and 0.357, respectively, indicating the DP model had the best explanatory power, while the PA model had the weakest (Table 8). The forest plot (Figure 1) visualized the regression results. Education level, annual income, and daily working hours significantly predicted all three burnout dimensions, with all 95% CIs excluding zero. Higher education level and annual income were negatively correlated with EE and DP but positively correlated with PA, whereas longer working hours increased EE and DP and decreased PA. In contrast, marital status, scheduling type, employment type, job position, and performance-related salary showed no significant effects on any dimension (all *p* > 0.05). After covariate adjustment, education level, annual income, and daily working hours remained stable independent influencing factors. The three burnout dimensions exhibited obvious heterogeneity. EE and DP shared identical influencing factors, whereas PA was poorly predicted by external demographic and work variables, suggesting it is primarily determined by latent psychological and organizational factors.

**Figure 1:**
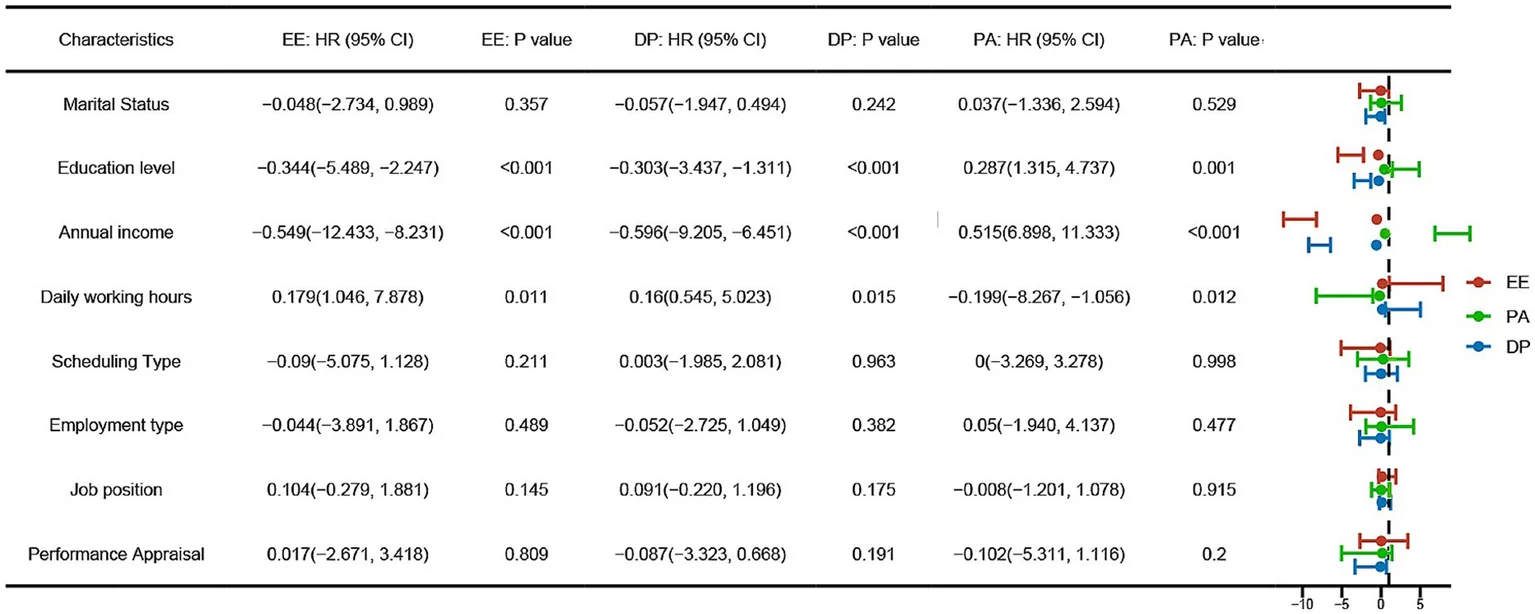
Forest plot of multiple linear regression analysis for influencing factors of job burnout dimensions.

**Table 8:** Overall performance of the model for job burnout dimensions.

Dimensions	*F* value	*p* value	Adjusted *R*^2^	Durbin-Watson
EE	25.559	<0.001	0.491	2.031
DP	32.220	<0.001	0.550	1.899
PA	15.144	<0.001	0.357	1.921

### 3.5 Multiple linear regression analysis of humanistic management perception

Four multiple linear regression models incorporating three-category job burnout were established for the four subdimensions of humanistic management perception (WCR, SCD, MSC, CVI). Model fitting indicators are listed in Table 9. All models were significant (*p* < 0.001) with Durbin-Watson values of 1.550–1.847 (no residual autocorrelation), and adjusted R^2^ ranged from 0.559 to 0.687. Forest plots (Figure 2 for WCR and SCD, Figure 3 for MSC and CVI) display standardized coefficients, 95% CIs and *p* values for all variables.

**Figure 2:**
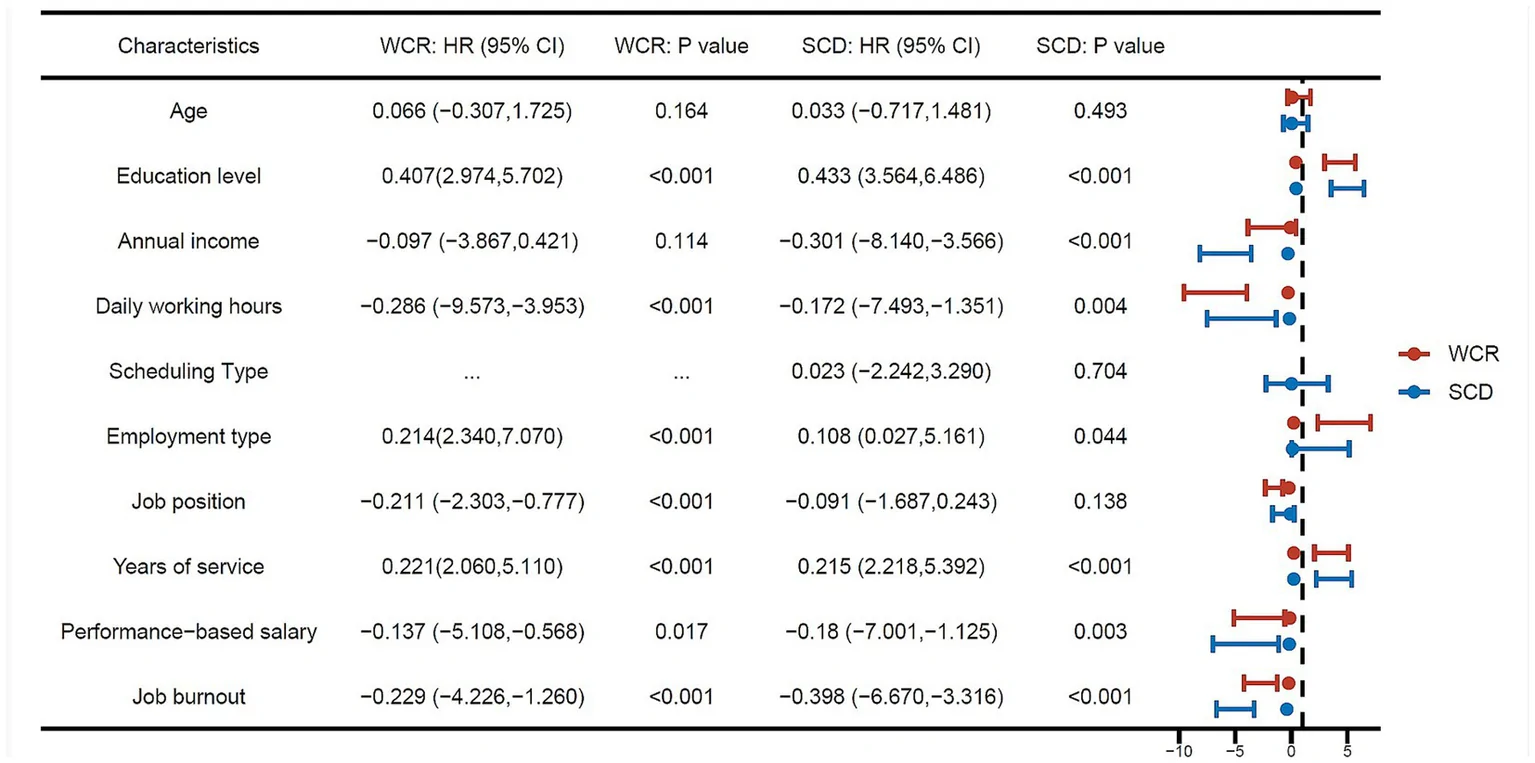
Forest plot of standardized regression coefficients for WCR and SCD.

**Figure 3:**
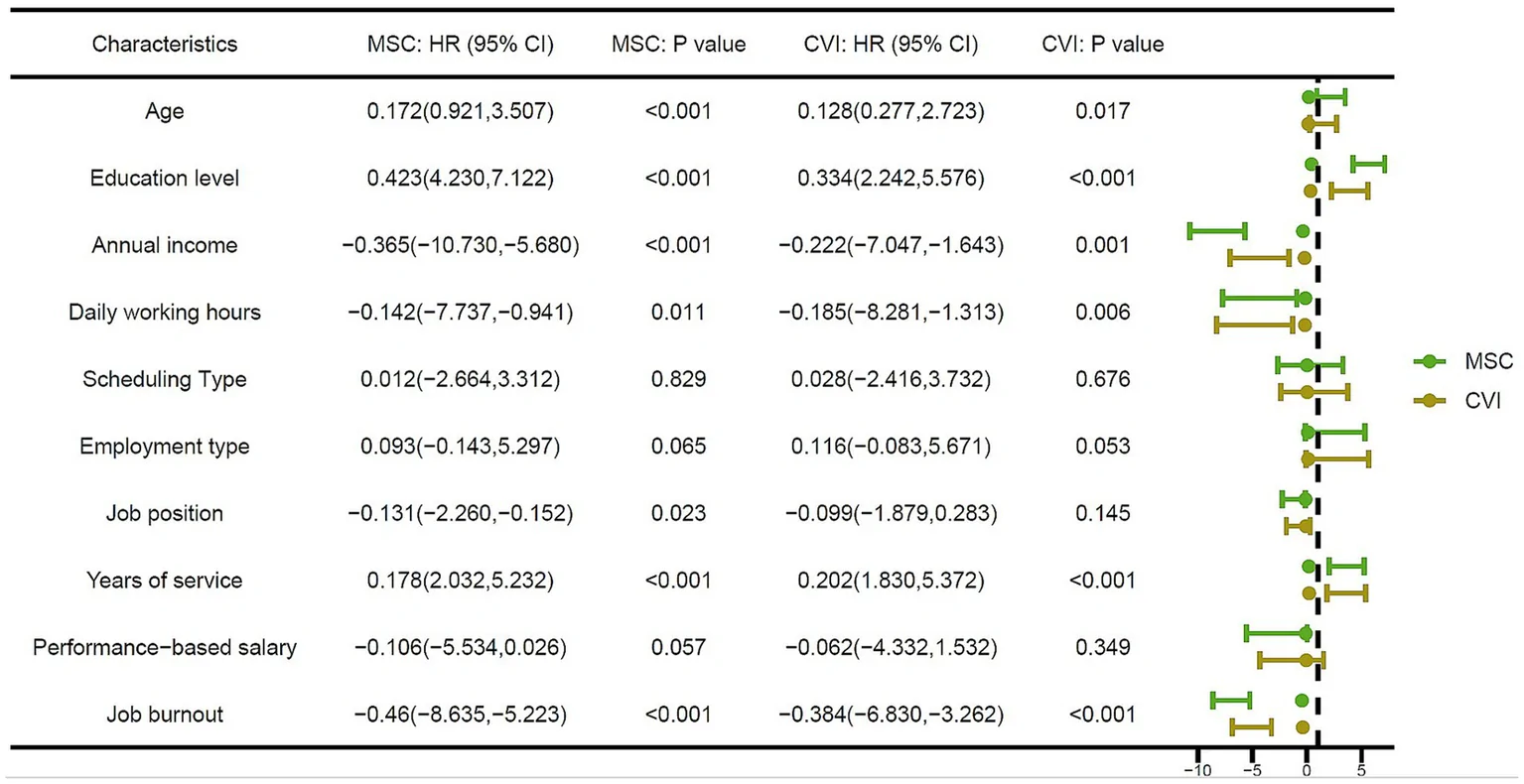
Forest plot of standardized regression coefficients for MSC and CVI.

**Table 9:** Overall performance of the regression models for humanistic management perception dimensions.

Dimensions	*F* value	*p* value	Adjusted *R*^2^	Durbin-Watson
WCR	39.413	<0.001	0.629	1.847
SCD	38.055	<0.001	0.645	1.702
MSC	45.839	<0.001	0.687	1.55
CVI	26.807	<0.001	0.559	1.843

After covariate adjustment, educational level, years of service, daily working hours and three-category job burnout were consistent independent influencing factors across all four dimensions. Higher education and longer tenure corresponded to higher perception scores, while longer working hours and greater burnout were linked to lower scores. Employment type affected WCR, SCD and CVI; age affected MSC and CVI; annual income affected SCD, MSC and CVI; job position affected WCR and MSC; performance-based salary affected WCR and SCD. Scheduling type showed no stable independent effects.

In brief, job burnout universally shapes staff humanistic management perception, and obvious heterogeneity exists across four subdimensions. The findings support targeted humanistic management strategies focusing on burnout mitigation.

### 3.6 Analysis of the correlations between the perception of humanistic management and job burnout

Pearson correlation analysis of 205 hospital support staff revealed significant correlations between all burnout indicators and subdimensions of perceived humanistic management (*p* < 0.01). The composite burnout score had moderate negative correlations with all four humanistic management subdimensions, among which CVI showed the strongest negative linkage to overall burnout. The results of the correlation analysis between occupational burnout (such as its dimensions) and each dimension of perceived humanistic management among hospital support staff are presented in Table 10.

**Table 10:** Correlation analysis of job burnout and its various dimensions with the various dimensions of perceived humanistic management among hospital support staff.

Dimensions	1	2	3	4	5	6	7	8
1. EE	1							
2. DP	0.880^**^	1						
3. PA	−0.819^**^	−0.727^**^	1					
4. Ahola standard scores	0.975^**^	0.930^**^	−0.893^**^	1				
5. WCR	−0.422^**^	−0.418^**^	0.354^**^	−0.429^**^	1			
6. SCD	−0.442^**^	−0.399^**^	0.376^**^	−0.438^**^	0.829^**^	1		
7. MSC	−0.481^**^	−0.437^**^	0.400^**^	−0.475^**^	0.838^**^	0.923^**^	1	
8. CVI	−0.490^**^	−0.471^**^	0.404^**^	−0.491^**^	0.789^**^	0.840^**^	0.869^**^	1

^**^p < 0.01.

## 4 Discussion

This study investigated the current status of job burnout and perceived humanistic management among hospital logistics support staff. Based on the theoretical link between organizational management and employees’ psychological status, we screened their independent influencing factors and explored the correlation and interaction between the two indicators. This research fills the research gap regarding hospital logistics support staff, and provides evidence-based and practical strategies to reduce burnout and optimize humanistic management for logistics teams.

In 1974, the American psychologist Freudenberger introduced the concept of occupational burnout. In 1981, Maslach and colleagues proposed a three-dimensional model of occupational burnout, which consists of emotional exhaustion (EE), depersonalization (DP), and reduced personal accomplishment (PA) (Kumar and Weil, 1998; Maslach and Jackson, 1981). Although occupational burnout spans various professions, it was first applied to and studied in health care workers, with pioneering research focusing on their symptoms and countermeasures (Obeng Nkrumah et al., 2025; Villalon Lopez et al., 2025; Rezuan et al., 2025; Pines and Maslach, 1978; Wang et al., 2025; Alsadaan, 2025; Mathew et al., 2025).

An integrative review revealed that the contributing factors of burnout among certified registered nurse anesthetists ranged from 12.5 to 72%, and the significant contributing factors to burnout included a lack of autonomy, leadership support, moral distress, poor relationships with physicians, the intention to leave one’s primary position, decreased job satisfaction, and fatigue (Congdon et al., 2025). A cross-sectional study of psychological burnout among health care professionals in a Malaysian public hospital revealed that the majority experienced personal and work-related burnout (81.9%), and significant associations were found between burnout and age (Rezuan et al., 2025). Unlike clinical staff who have been widely studied, hospital logistics personnel perform repetitive and high-intensity auxiliary work. This group has long received inadequate organizational attention, placing them at high risk of job burnout. In this study, univariate analyses indicated that demographic characteristics, working conditions and occupational attributes were significantly correlated with the three dimensions of job burnout. Subsequent multiple linear regression analyses identified educational attainment, annual income and daily working hours as consistent independent influencing factors across all burnout subdimensions. Higher educational level and annual income were linked to lower scores on EE and DP, as well as higher PA scores. By contrast, longer daily working hours exacerbated emotional exhaustion and depersonalization, and diminished personal accomplishment. After controlling for confounding variables, marital status, work schedule type, employment category, job position and performance appraisal exerted no significant independent effects on job burnout.

Daily working hours, as a core work characteristic, exerted a consistent adverse impact on job burnout. Multiple international studies have verified that prolonged working hours are a key occupational risk factor for burnout among all types of healthcare staff (Lin et al., 2021; Amiri et al., 2024). Excessively long working hours continuously consume employees’ emotional and physical resources, which aligns with the Conservation of Resources theory: sustained work resource depletion inevitably triggers burnout symptoms (Lin et al., 2021). Annual income and educational attainment reflected employees’ economic status and comprehensive quality. Reasonable income distribution can improve staff recognition and reduce negative work emotions, while employees with higher educational levels tend to have stronger psychological adjustment ability and clearer career cognition, thus maintaining a better psychological state at work (Johnson et al., 2020). Global meta-analysis also indicated that unfair salary systems and inadequate welfare are important risk factors for healthcare worker burnout (Amiri et al., 2024). Given the occupational characteristics of hospital logistics support staff, it is impractical to take improving academic qualifications as a primary intervention measure. Instead, hospitals should prioritize optimizing work scheduling, controlling daily working hours reasonably and improving the salary and welfare system to cut down burnout risks from the source.

Humanistic management is a people-oriented organizational management mode, and employees’ perceived experience of its implementation directly reflects the effectiveness of hospital management. Consistent with the research objective, this study further analysed the status and influencing factors of humanistic management perception among logistics support staff. After incorporating three-category burnout as a covariate into multiple linear regression models, the results demonstrated that educational attainment, years of service, daily working hours and burnout level were universal independent influencing factors across all four subdimensions of humanistic management perception. Higher educational level and longer working tenure improved staff’s humanistic management perception, whereas longer daily working hours and higher burnout levels significantly reduced perception scores. In addition, age, annual income, employment type, job position and performance-based salary only produced independent effects on partial subdimensions, indicating obvious heterogeneity in influencing factors among different dimensions. Similar heterogeneity of influencing factors was also reported in research on hospital administrative and support staff’s occupational psychology (Lin et al., 2025).

The core focus of this study lies in unpacking the interactive association between perceived humanistic management and job burnout. Pearson correlation analysis confirmed that every subdimension of humanistic management perception was significantly negatively correlated with overall burnout (*p* < 0.001), and the total composite scores of the two constructs also yielded a robust negative association. Combined with multiple linear regression findings, burnout served as an independent predictor across all four humanistic subdimensions, which solidifies the tight reciprocal interplay between the two variables.

Mechanistically, sound humanistic management can effectively mitigate job burnout in multiple ways. First, humanistic management builds a respectful and supportive organizational atmosphere, provides effective emotional support for staff, relieves emotional exhaustion caused by long-term work pressure, and supplements individual psychological resources. Existing studies have confirmed that positive workplace support and empathetic management can significantly reduce burnout levels of healthcare workers. Second, standardized humanistic systems, fair management modes and positive organizational culture can enhance employees’ sense of organizational belonging and value identity, and avoid the emergence of depersonalization and cynical work attitudes. A study on medical and non-medical healthcare personnel pointed out that organizational justice, as an important part of humanistic management, is negatively correlated with burnout severity. Third, humanistic management focusing on staff development can improve employees’ work experience and sense of achievement. Conversely, severe burnout will also damage employees’ subjective perception of organizational humanistic care: when staff are trapped in long-term emotional exhaustion and low work engagement, they cannot effectively perceive the hospital’s humanistic measures, forming a mutually restrictive interactive state. Leadership behavior and organizational humanistic atmosphere have also been proven to be core moderators between work status and psychological feelings of hospital employees.

In view of the above correlation and mechanism, hospital management should take targeted organizational improvement measures rather than focusing on changing individual demographic characteristics. First, scientifically optimize post allocation and working time arrangement, strictly control excessive daily working hours, and reduce work resource consumption of logistics staff. Second, improve the salary distribution and welfare system, balance the income level of different posts, and enhance employees’ work satisfaction. Third, implement differentiated humanistic management according to dimensional heterogeneity: strengthen work communication and post care for front-line logistics posts, and optimize internal management systems and organizational culture. Fourth, establish regular burnout screening and dynamic psychological intervention mechanisms to block the adverse interactive cycle between burnout and poor management perception. Relevant intervention studies have confirmed that systematic organizational support and psychological counseling are effective ways to improve the psychological status of healthcare auxiliary staff.

This study has multiple strengths including a unique non-clinical sample of hospital logistics staff, standardized statistical methods and reliable measurement tools, and it proposes practical management strategies supported by international literature. Nevertheless, its single-center cross-sectional design, limited sample coverage and incomplete variable selection restrict the generalizability and causal inference of the conclusions. Future multi-center longitudinal studies and relevant intervention trials are recommended to clarify the causal relationship between humanistic management perception and job burnout.

## 5 Conclusion

In summary, this study adds empirical data on occupational mental health for underrepresented hospital logistics staff. For hospital administrators, curbing excessive daily workload, establishing fair compensation schemes, and delivering personalized humanistic support can significantly boost staff’s positive organizational perceptions and reduce burnout. Constrained by its single-center cross-sectional design and limited variable selection, multi-center longitudinal trials and intervention research are required to further clarify the causal relationship between perceived humanistic management and job burnout.

## Data Availability

The original contributions presented in the study are included in the article/supplementary material, further inquiries can be directed to the corresponding authors.
